# Eggshell Membrane-Templated MnO_2_ Nanoparticles: Facile Synthesis and Tetracycline Hydrochloride Decontamination

**DOI:** 10.1186/s11671-018-2679-y

**Published:** 2018-08-28

**Authors:** Qi Wang, Chunlei Ma, Jianke Tang, Cuihong Zhang, Lihua Ma

**Affiliations:** 1 Chemistry and Chemical Engineering Department, Taiyuan Institute of Technology, Taiyuan, 030008 Shanxi China; 2 NMR and Drug Metabolism Core, Baylor College of Medicine, One Baylor Plaza, Houston, TX 77030 USA; 3 College of Science and Engineering, University of Houston-Clear Lake, 2700 Bay Area Blvd, Houston, TX 77058 USA

**Keywords:** MnO_2_ nanoparticles, Eggshell membrane, Tetracycline, Decontamination

## Abstract

Taking advantages of reticular proteins and reductive groups on the surface, eggshell membrane (ESM) was selected to synthesize MnO_2_ nanoparticles from potassium permanganate through a super simple way in which ESM acted as both template and reductant. This process avoided harsh reaction conditions or complicated aftertreatments and thus owned the merits of green synthesis, handy operation, low cost, and easy purification. The ESM-templated MnO_2_ nanoparticles (MnO_2_ NPs/ESM) were characterized, and the content of nanomaterials on the template was tested. MnO_2_ NPs/ESM showed a good capacity for decontamination of tetracycline hydrochloride (TCH). The macroscopical materials can be separated easily by taking the membrane out to stop the degradation instead of centrifugation or filtration. It was studied that 72.27% of TCH (50 mg/L) was decontaminated in 20 min by 0.1920 g/L MnO_2_ nanoparticles, and removal efficiency could reach 83.10% after 60 min under buffered condition. The kinetics was studied with or without buffer, and it was concluded that the degradation process followed a pseudo-second-order model. The facile synthesis of materials and effective degradation would facilitate the nano-MnO_2_-based decontamination applications.

**Electronic supplementary material:**

The online version of this article (10.1186/s11671-018-2679-y) contains supplementary material, which is available to authorized users.

## Background

Pharmaceuticals and personal care products (PPCPs) are a kind of emerging water pollutions and are concerned closely by researchers in consideration of ecology and human health [1–5]. Antibiotics as a medicine to treat and prevent bacterial infections are used worldwide, accompanying with which upsetting risks to the environment have gradually appeared [6]. As a representative, tetracycline (TC) medicines have been used in veterinary science and aquaculture for years [7]. However, TCs can hardly be degraded in the environment and thus persist for a long time [8, 9], which lead to a various negative influence on the ecosystem or human health [10–13]. Therefore, screening a facile and effective way to decontaminate TC-contaminated water has become a hotspot of research. One promising technique may be the assistance of manganese dioxide nanomaterials.

Manganese dioxide nanomaterials have been extensively studied owing to their unique merits of high surface area, tunable structure, catalytic oxidation activity, and eco-harmless [14, 15]. Therefore, nano-MnO_2_-based applications have covered various fields ranging from catalysis [16, 17], sensors [18, 19], and capacitors [20, 21] to drug delivery [22, 23] and cancer therapy [24, 25]. By the same token, MnO_2_ nanomaterials with adsorption and oxidation properties have applied to wastewater treatment. Water pollutants including heavy ions [26], organic dyes [27], and phenols [28] treated by MnO_2_ nanomaterials were reported. Meanwhile, antibiotics such as levofloxacin [29], ciprofloxacin [30], norfloxacin [31], sulfamethoxazole [32], sulfadiazine [33], cefazolin [34], lincosamide [35], and TCs [36, 37] have been successfully decontaminated through MnO_2_ treatment. Specific to TC antibiotics, highly porous MnO_2_ nanosheets were utilized to degrade tetracycline, and pH, temperature, and dose-based kinetics were investigated [38]. A MnO_2_-based scheme was applied to remove tetracycline hydrochloride (TCH) and As(III) simultaneously, and the interactive effect on arsenic and antibiotics during MnO_2_ treatment was studied [39]. Degradation of tetracycline antibiotics by MnO_2_ was performed, and transformation kinetics and pathways were reported [40]. Though high removal efficiency of TCs was obtained in the abovementioned works, however, the degradation operation usually involved in the centrifugation or filtration in order to separate the material from antibiotics solutions, which took much of the treatment time and made the process complicated.

Eggshell membrane (ESM) as a unique biomaterial with extraordinary properties has been utilized in materials science extensively [41]. The main composition of fiber in ESM is a protein which endows ESM the ability to bind metal. Noble metal nanomaterials like Ag NPs and Au NPs were successfully synthesized using ESM as a template [42–44]. In addition, metal oxide nanomaterials such as ZnO [45], Co_3_O_4_ [45], PbO [45], Mn_3_O_4_ [46], and TiO_2_ [47] were also prepared through ESM templating, which made the synthesis facile and under control and therefore provided a novel path for the synthesis of metal or metal oxide nanoparticles.

In this work, eggshell membrane-templated MnO_2_ nanoparticles (MnO_2_ NPs/ESM) were synthesized simply and quickly by a bio-templating method. Eggshell membrane played dual roles as a template and a reductant making nanoparticles dispersed uniformly on the macroscopical membranes. Combining the oxidizing MnO_2_ nanoparticles with the easy-to-manipulate membrane, MnO_2_ NPs/ESM were further applied to tetracycline hydrochloride decontamination, in which nanomaterials could be separated easily by simply taking out of solutions.

## Methods

### Materials and Apparatus

Deionized water with a conductivity of 18.2 MΩ cm^−1^ was used in this experiment from a water purification system (ULUPURE, Chengdu, China). Potassium permanganate (KMnO_4_, *M*_*w*_ = 158.03), MnO_2_ powder, and other reagents were at least of analytical grade and purchased from Kemiou Chemical Co. Ltd. (Tianjin, China). Tetracycline hydrochloride (TCH, USP grade) and glutathione (GSH, 98%) were purchased from Aladdin Reagents Company (Shanghai, China). Eggshell membrane (ESM) was peeled off carefully from a fresh eggshell which is obtained from Hongye student mess hall of Taiyuan Institute of Technology. PBS buffer solutions (0.2 M, pH = 7.0) were prepared by mixing 39 mL NaH_2_PO_4_ solution (0.2 M) and 61 mL Na_2_HPO_4_ solution (0.2 M), and PBS solutions with different pH values were prepared by titrating the abovementioned solution with sodium hydroxide or hydrochloric acid solution (both concentrations were 0.2 M) to the required pH values.

Scanning electron microscopy (SEM) of MnO_2_ NPs/ESM was carried out on a Quanta 200 FEG scanning electron microscope for the morphology observation. Transmission electron microscopy (TEM) and high-resolution transmission electron microscopy (HRTEM) of MnO_2_ NPs were performed on a Tecnai-G20 transmission electron microscope. The size distribution of as-prepared MnO_2_ NPs was obtained at a laser particle sizer (Malvern Nano-ZS90). X-ray photoelectron spectroscopy (XPS) was collected on an AXIS ULTRA DLD electron spectrometer (Kratos) with monochromatic Al Kα radiation for the surface composition and chemical state test of the product. Thermogravimetry (TG) analysis of ESM and MnO_2_ NPs/ESM was measured in air at a heating rate of 10 °C/min on a Rigaku TG thermal analyzer (Rigaku Co. Japan). Fourier transform infrared spectroscopy (FTIR) from 4000 to 400 cm^−1^ of ESM and MnO_2_ NPs/ESM was recorded in KBr discs on a Tensor II FTIR spectrometer (Bruker, Germany), and the spectra were processed through deconvolution. The ultraviolet-visible (UV-vis) absorption spectra of TCH were recorded on a TU-1901 UV-vis spectrophotometer (Puxi, China).

### Synthesis of ESM-Templated MnO_2_ Nanoparticles

The eggshell membrane-templated MnO_2_ nanoparticles (MnO_2_ NPs/ESM) were synthesized through a straightforward and facile method. In a typical process, the eggshell membrane was firstly peeled off from a fresh eggshell manually and washed ten times with deionized water to remove the needless egg white. After drying under room temperature, the clean ESM was then cut into slices with 5 mg weight each. Upon synthesis, ten slices of ESM were soaked into 20 mL KMnO_4_ solution (1 mmol/L) and the open system kept stirring under room temperature. Thirty-five minutes later, the ESM slices were taken out and washed ten times with deionized water to remove the redundant solution. At last, the obtained membranes were dried and stocked at room temperature for further characterization and use.

### Decontamination of TCH

The decontamination of TCH was performed by adding MnO_2_ NPs/ESM into the TCH solutions simply and stirred under room temperature. Twenty slices of MnO_2_ NPs/ESM were placed into 15 mL TCH solutions (50 mg/L) which were diluted by PBS buffer solutions and kept stirring for 60 min. The UV-vis spectra of TCH solutions after treatment were recorded immediately at room temperature. All of the absorption intensities of TCH measurement were set at a wavelength of 358 nm. The removal efficiency (*R*, %) was calculated using the equation below:

$$ R=\frac{C_0-C}{C_0}\times 100\% $$

where *C*_0_ and *C* (mg/L) stand for the initial and final concentrations of TCH in the treatment solutions, respectively.

## Results and Discussion

### Mechanism and Monitoring of MnO_2_ NPs/ESM Synthesis

The synthesis of MnO_2_ NPs/ESM was performed in an open system with ESM as biotemplate. The eggshell membrane was composed of many fibrous proteins on which lots of reductive groups like –OH, –NH_2_, –SH, etc. were interspersed. An in situ redox reaction was triggered once the KMnO_4_ was introduced. While MnO_2_ was generated, it grew gradually around these active groups. As a consequence, it was dispersed uniformly on the surface of fibrous proteins to form ESM-templated MnO_2_ NPs.

**Fig. 1:**
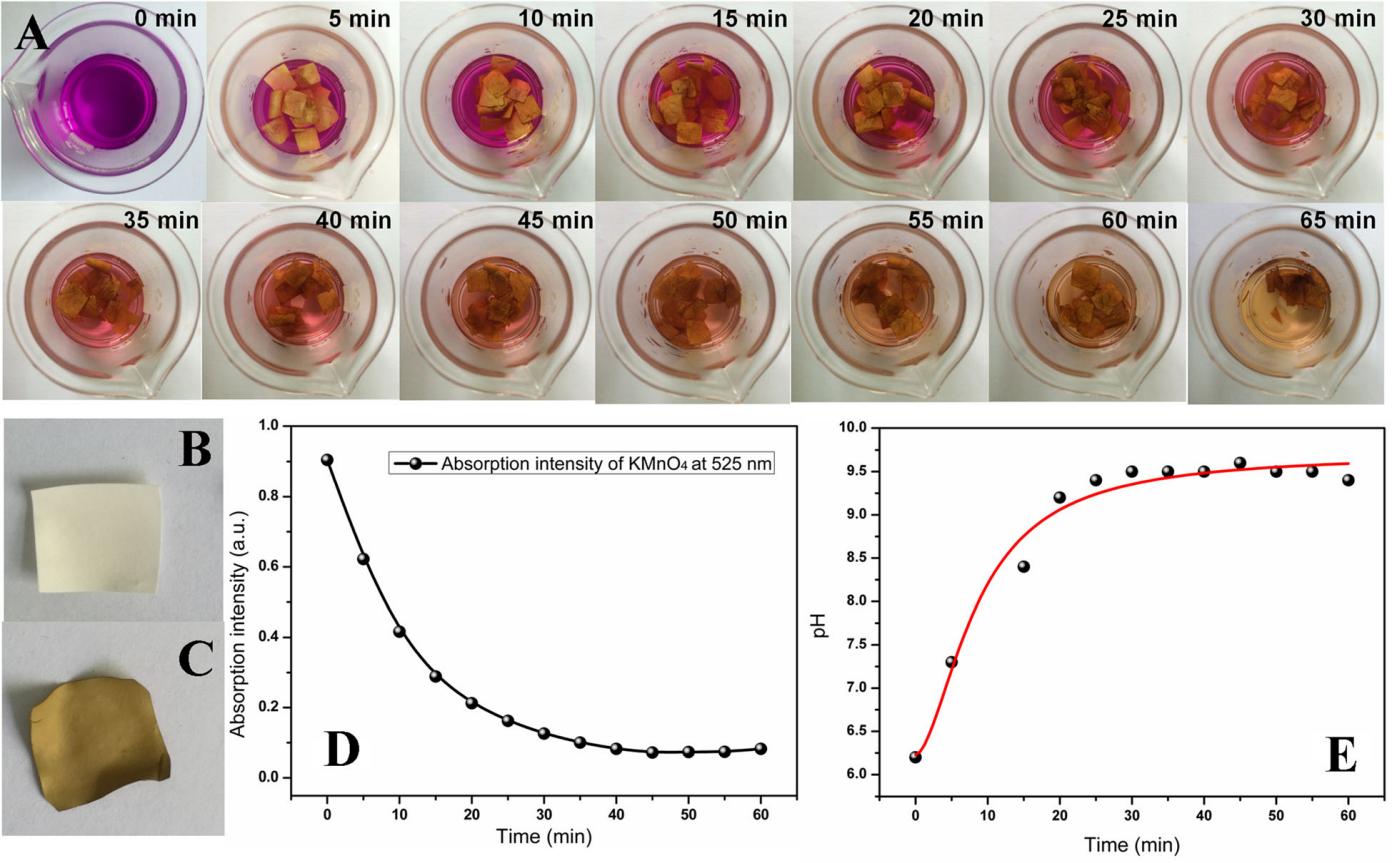
**a** The photos of the synthetic system at different times. **b** The image of ESM slice before the redox reaction. **c** The image of MnO_2_ NPs/ESM. **d** The absorption intensity of KMnO_4_ at 525 nm. **e** pH of the synthetic system at different times

Figure 1a displayed the photos of synthesis system at different times, in which purple KMnO_4_ solution turned into light brown gradually, and meanwhile, the white ESM slices became brown (Fig. 1b, c). To monitor the synthetic process, the absorption intensity of KMnO_4_ at 525 nm and pH of this system were investigated in Fig. 1d, e. As shown, the absorption intensity of KMnO_4_ decreased with time prolonged, and pH, conversely, improved gradually. Two sets of data both showed a platform after 35 min, and therefore, the synthesis time was selected. The increasing of pH was attributed to the formation of –OH during the reaction and a reaction course was speculated below:

ESM (Red) + KMnO_4_ + H_2_O → MnO_2_/ESM (Ox) + OH¯ + K^+^

### Characterization of MnO_2_ NPs/ESM

**Fig. 2:**
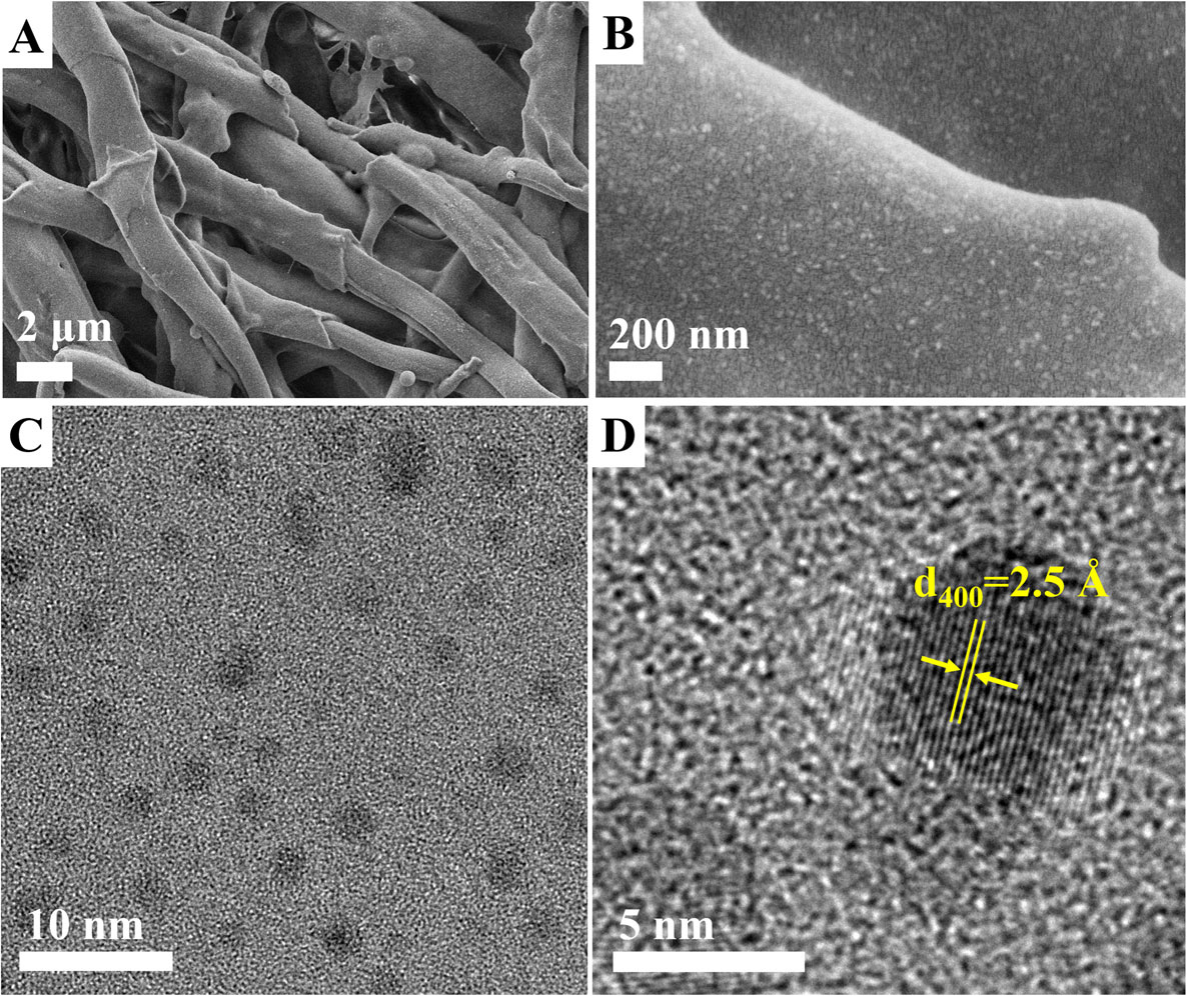
The SEM images of MnO_2_ NPs/ESM with different scale bars (2 μm (**a**) and 200 nm (**b**)). The TEM (**c**) and HRTEM (**d**) images of MnO_2_ NPs, the scale bars were 10 nm and 5 nm, respectively

The morphology of the obtained MnO_2_ NPs/ESM was investigated by scanning electron microscopy (SEM) in Fig. 2. Multilayered and intersecting fibrin network was observed in Fig. 2a, b. After further amplification, lots of particles were found uniformly coated on the surface of fibrous proteins. Therefore, it was concluded that ESM acted not only as a reductant but also a template during the synthesis. To further investigate the size of MnO_2_ particles, a laser particle sizer test was carried out. In order to rule out the possibility that the particles with 4.8 nm were decomposed proteins, MnO_2_ NPs/ESM and equal amounts of blank ESM (control) were first placed in NaOH solutions (0.1 M) and boiled for 30 min and then filtered to form solutions to meet the test condition. It was found in Additional file 1: Figure S1 that the average size of MnO_2_ NPs was 4.8 nm. The photos of MnO_2_ NPs/ESM before and after NaOH treatment were displayed in Additional file 2: Figure S2A. It was obvious that the brown color of the membrane faded evidently while the membrane kept unchanged after NaOH treatment, indicating that the MnO_2_ NPs were released from the template. In thinking about the problem that the size of the protein from the eggshell may interfere with the results, the filtrated solutions after NaOH treatment were obtained from both blank ESM and MnO_2_ (Additional file 2: Figure S2B) to be colorless and brown, respectively. In addition, the size distribution data of ESM after NaOH treatment displayed an average size of 1.7 nm in Additional file 2: Figure S2C. Therefore, the possibility that the particles with 4.8 nm were decomposed proteins from ESM itself was ruled out. Based on this, TEM was captured after the aforementioned filtrate was dialyzed. As shown in Fig. 2c, spherical nanoparticles were observed and the size was consistent with the one in Additional file 1: Figure S1. The HRTEM image in Fig. 2d indicated a lattice spacing of 2.5 Å that was well coincident with the (400) lattice plane of α-MnO_2_ [48].

**Fig. 3:**
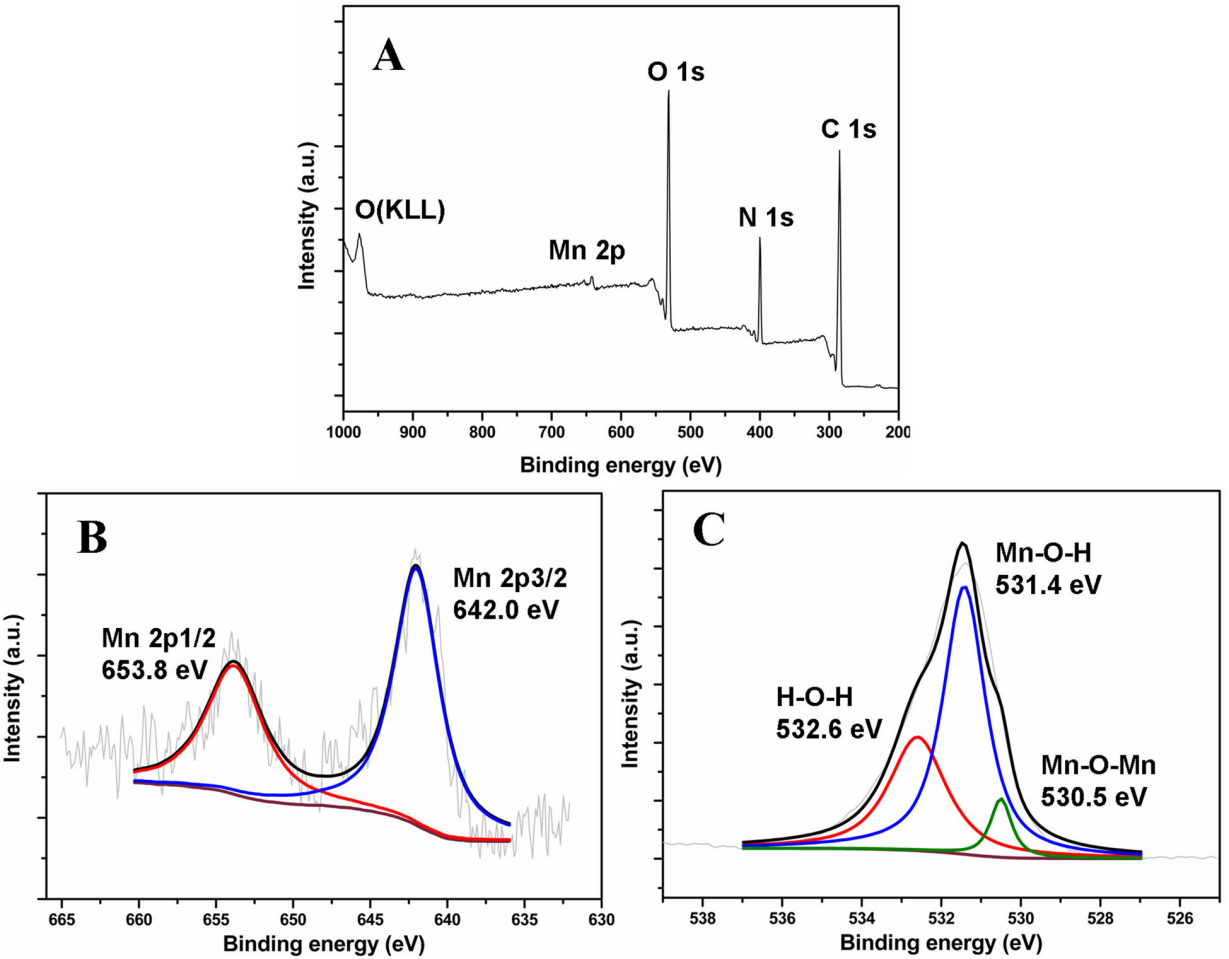
The XPS (**a**) full scan, (**b**) Mn 2p, (**c**) O 1s spectra of as-prepared MnO_2_ NPs/ESM

Besides, X-ray photoelectron spectroscopy (XPS) technique was carried out for the surface composition and elemental analysis of the obtained MnO_2_ NPs/ESM. The full-scan spectrum (Fig. 3a) indicated that the synthesized material was composed of elements Mn 2p, O 1s, N 1s, and C 1s. Element C 1s, N 1s, and partial O 1s came from the template ESM. The partial XPS spectra of Mn 2p and O 1s were measured to study the details. As shown in Fig. 3b, two peaks at 653.8 and 642.0 eV can be assigned to Mn 2p_1/2_ and Mn 2p_3/2_, respectively. The O 1s spectrum (Fig. 3c) can be divided into three component peaks with binding energy at 532.6, 531.4, and 530.5 eV, which were attributed to H–O–H, Mn–O–H, and Mn–O–Mn, respectively. The above data demonstrated that the as-prepared material was ESM-templated MnO_2_ NPs.

To further verify this result, GSH solution was applied to the test thus obtained material inspired by a special reaction between GSH and MnO_2_ [49, 50]. As shown in Additional file 3: Figure S3, the brown color of MnO_2_ disappeared after soaking into GSH solution for 1 min, indicating that the materials coated on ESM were MnO_2_. Furthermore, thermogravimetry (TG) analysis was carried out to measure the mass content of MnO_2_ on ESM. The black and red curves in Additional file 4: Figure S4 stood for the mass changes of ESM only and MnO_2_ NPs/ESM, respectively. The relative quality of ESM was almost zero at 600 °C indicating that ESM was totally burnt out. However, the relative quality of ESM-templated MnO_2_ NPs remained at 2.61% after ESM was burnt out. It was reported that MnO_2_ was thermally decomposed at 500 °C and conversed into Mn_2_O_3_ [51]. Moreover, further thermal decomposition of Mn_2_O_3_ to Mn_3_O_4_ occurred above 1000 °C [52]. Therefore, the mass content of 2.61% at 800 °C obtained in this experiment reflected the content of Mn_2_O_3_. According to the mass conservation of Mn, the original MnO_2_ content loading on the ESM was calculated to be 2.88%.

FTIR spectra (Additional file 5: Figure S5) of ESM and MnO_2_ NPs/ESM were collected after grinding the materials into powder. The interactions between proteins and nanoparticles mainly involve secondary structure changes, which are reflected on the band of amide I ~ 1650 cm^−1^ (which may shift a bit) or amide II ~ 1550 cm^−1^. However, there were no obvious changes of peak position around 1650 or 1550 cm^−1^ of ESM before and after MnO_2_ was involved, which was different from the previously reported results that could demonstrate the structural change of protein [53]. In order to get into the details and avoid missing any minor changes, deconvolution was applied to these spectra. Even though no observable peaks were shown up around 1650 or 1550 cm^−1^, a new peak at 506 cm^−1^ appeared after MnO_2_ NPs loading, and it was associated with the characteristic vibrational mode of Mn–O [54].

Mn has various oxidation states, so there are a few types of oxides, such as Mn_2_O_3_, MnO, and MnO_2_. The binding energy of Mn_2_O_3_ is close to that of MnO_2_. In order to examine the oxidation state of Mn in this work, the HRTEM of as-prepared materials was imaged and displayed in Fig. 2d. The lattice spacing of 2.5 Å detected is well coincident with the (400) lattice plane of α-MnO_2_ [48]. Moreover, our Mn materials were obtained based on the redox reaction between KMnO_4_ and ESM under the neutral condition that favored the formation of MnO_2_ instead of other oxidation states [55]. Importantly, as-prepared materials possess the reaction activity with GSH (Additional file 3: Figure S3), which is also a testimonial that the nanoparticle is MnO_2_ [49, 50]. It was also reported that MnO_2_ could thermally be decomposed at 500 °C and conversed into Mn_2_O_3_ [51]. The TG curve of the as-prepared materials in Additional file 4: Figure S4 shows an obvious weight loss around 500 °C, indicating the transformation from MnO_2_ to Mn_2_O_3_, which is another testimony that the oxidation state of Mn is MnO_2_.

### TCH Decontamination by MnO_2_ NPs/ESM

**Fig. 4:**
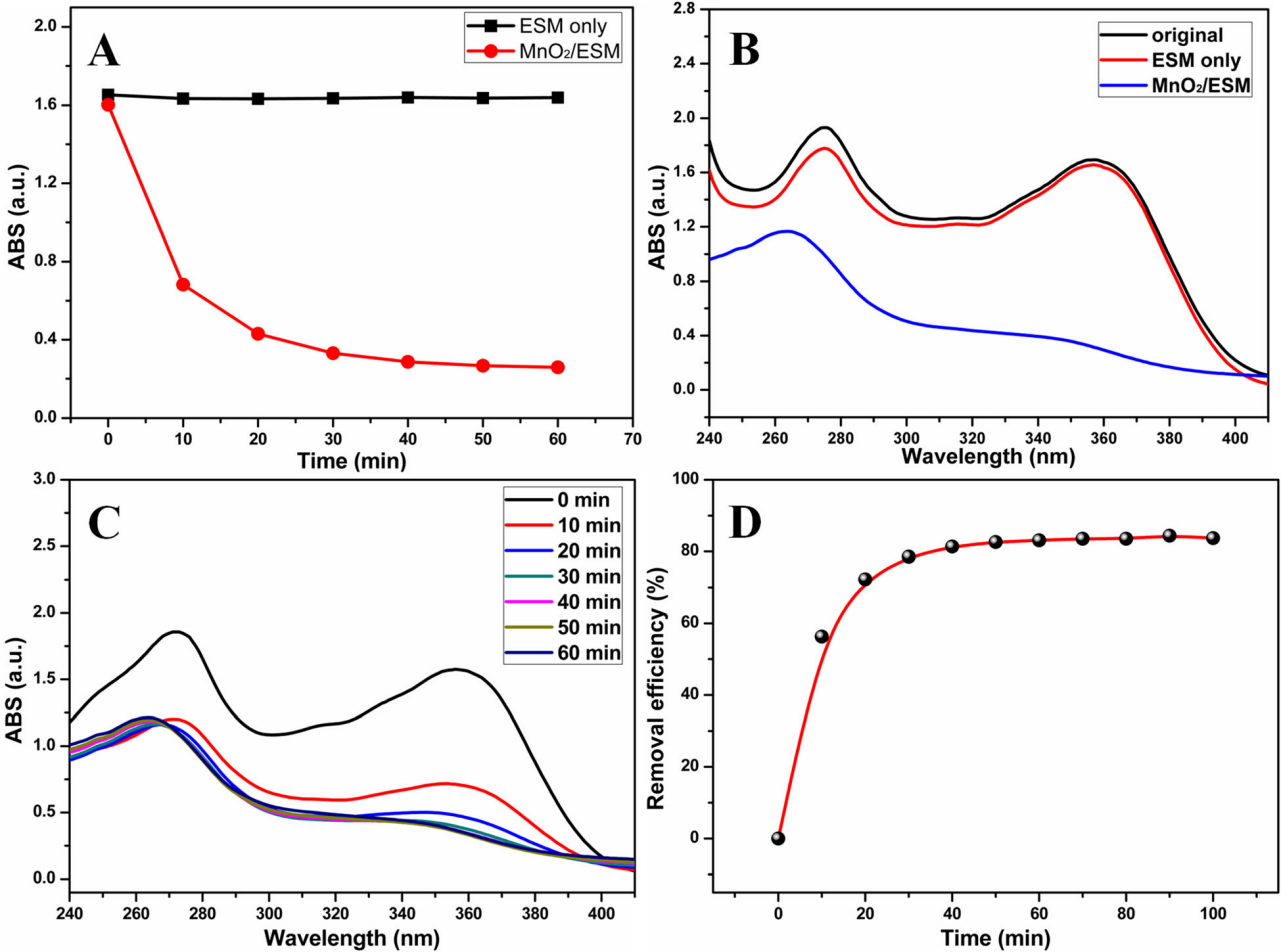
**a** The time-dependent absorption intensity of TCH by ESM and MnO_2_ NPs/ESM treatment. **b** The UV-vis absorption spectra of TCH before and after ESM or MnO_2_ NPs/ESM treatment. **c** The time-dependent UV-vis absorption spectra of TCH and (**d**) removal efficiency treated by MnO_2_ NPs/ESM. (Conditions: 20 slices of MnO_2_ NPs/ESM or ESM, the initial concentration of TCH was 50 mg/L, controlled pH was 3.0)

Taking advantages of oxidative MnO_2_ NPs and macroscopical template, MnO_2_ NPs/ESM were applied to tetracycline hydrochloride (TCH) decontamination owing to the effective removal and easy operation. Figure 4a displayed the time-dependent absorption intensity of TCH at 358 nm treated by ESM only (black) and MnO_2_ NPs/ESM (red). It was shown that absorption intensity kept unchanged in the presence of ESM only. However, it dropped sharply first and flattened out gradually under MnO_2_ NPs/ESM treatment. This evident contrast demonstrated the capacity of MnO_2_ NPs/ESM for TCH decontamination. Similarly, the UV-vis absorption spectra of TCH after ESM treatment hardly changed, but the absorption peak at 358 nm decreased obviously after MnO_2_ NPs/ESM decontamination (Fig. 4b). Figure 4c investigated the absorption spectra variation of TCH, in which the absorption peak at 270 nm lowered in the first 10 min, but another peak at 358 nm decreased along with time was observed. The time-dependent removal efficiency by MnO_2_ NPs/ESM decontamination was calculated in Fig. 4d, and it was found that removal efficiency was 72.27% at 20 min and it can reach 83.10% in 60 min.

### Effect of pH and Buffer on TCH Decontamination

**Fig. 5:**
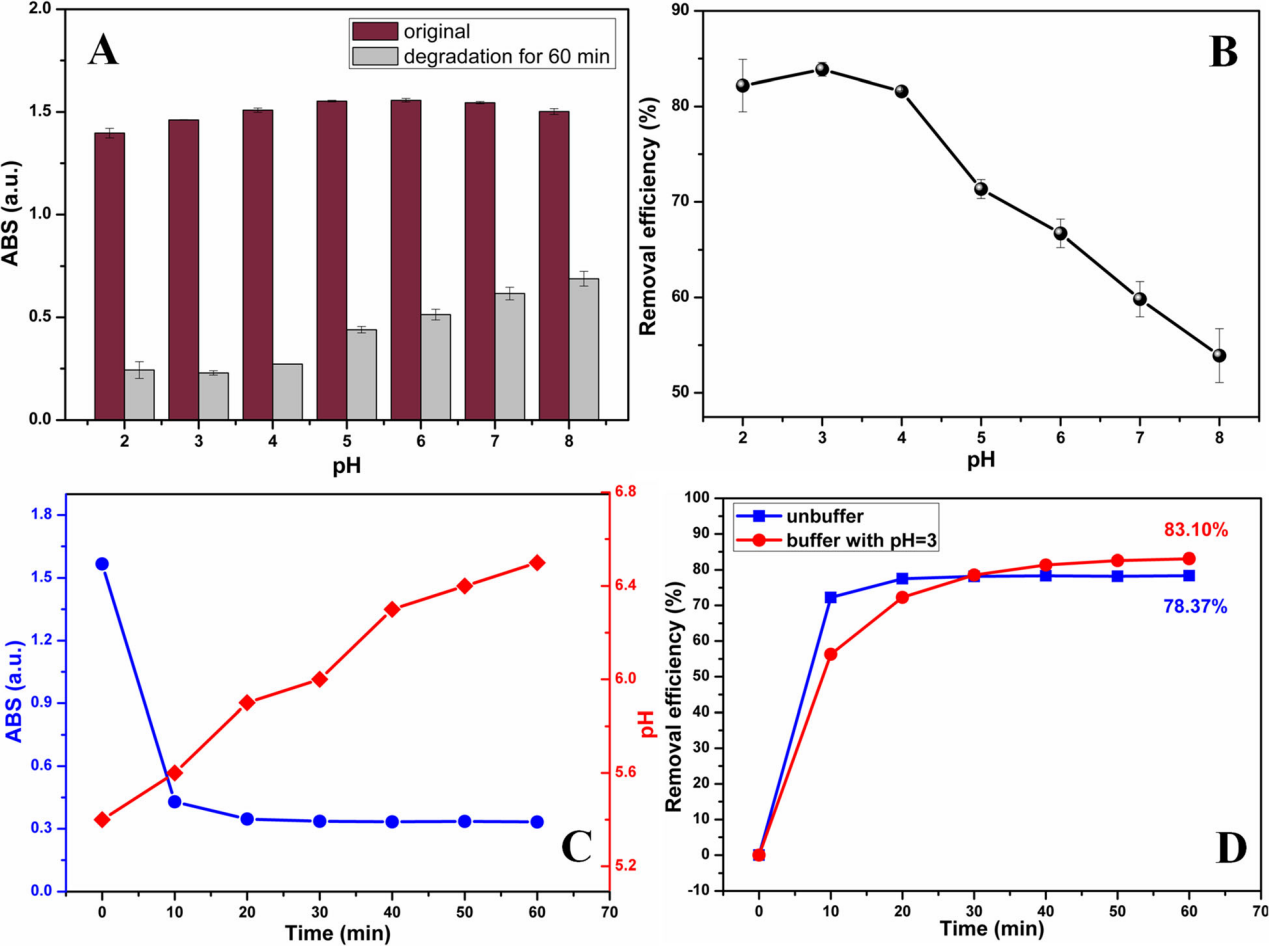
**a** The absorption intensity of TCH before and after degradation and (**b**) removal efficiency under different pH. **c** The time-dependent absorption intensity of TCH and pH variation under unbuffered condition. **d** Comparison of removal efficiency of TCH under buffered and unbuffered conditions. (Conditions: 20 slices of MnO_2_ NPs/ESM, the initial concentration of TCH was 50 mg/L.)

The pH played an important role in MnO_2_-based oxidative degradation, and the effect of pH on TCH decontamination in this work was investigated. Figure 5a displayed the absorption intensity of TCH before and after MnO_2_ NPs/ESM treatment for 60 min under different pH, and the corresponding removal efficiency was calculated in Fig. 5b. It was demonstrated that the optimal decontamination of TCH by MnO_2_ NPs/ESM was achieved under PBS buffer with a pH of 3.0. Moreover, TCH decontamination by MnO_2_ NPs/ESM without buffer solution was investigated in Fig. 5c, in which the absorption intensity of TCH decreased gradually and pH of the degradation system was steadily improved. The same phenomenon of pH increase during the decontamination process was also reported in a previous work [38]. It was worth noting that the removal efficiency without buffer increased more rapidly than that under a buffered condition at the beginning (first 20 min). Then as the time passed, however, the removal efficiency with buffer exceeded the one without buffer after 30 min (83.10% for buffered and 78.37% for the unbuffered condition at 60 min). Removal efficiencies were monitored  through concentration variations of TCH which were calculated from linear calibration plot (Additional file 6: Figure S6 and Additional file 7: Figure S7). Under the buffered condition, saline ions from PBS hindered the diffusion of TCH molecules onto the surface of MnO_2_ NPs for further reaction, and hence, the reaction rate was lower than that of without a buffer. However, pH increase of reaction system along with time under unbuffered condition limited the oxidative capacity of MnO_2_ NPs, and thus, the removal efficiency cannot reach the one obtained under controlled optimal pH.

### Kinetic Study of TCH Decontamination

**Fig. 6:**
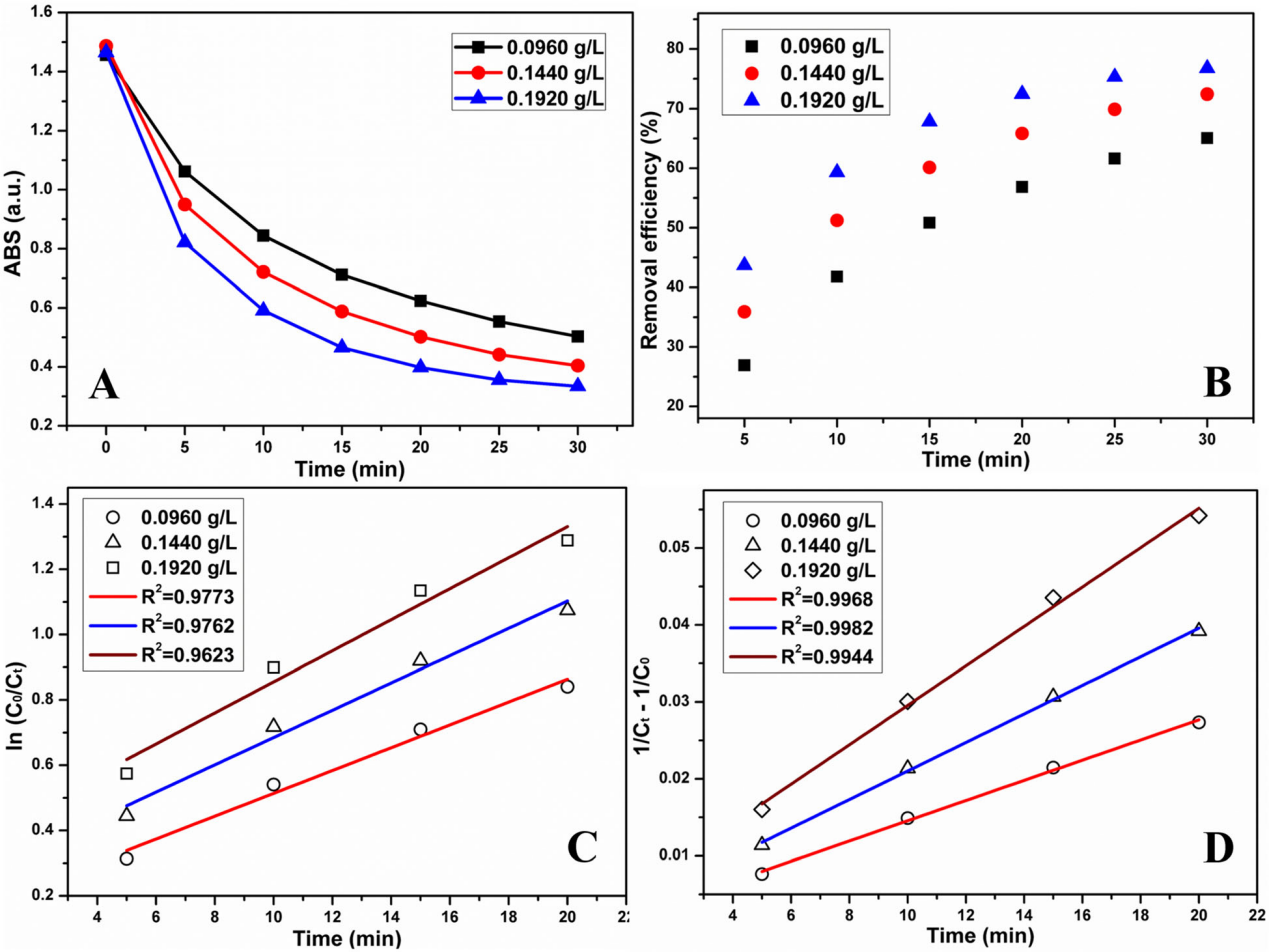
The time-dependent **a** absorption intensity of TCH and **b** removal efficiency by different amounts of MnO_2_ NPs/ESM treatment. **c** Linear first-order kinetic plots and **d** linear second-order kinetic plots by different amounts of MnO_2_ NPs/ESM treatment. (Conditions: initial concentration of TCH was 50 mg/L, controlled pH was 3.0)

**Fig. 7:**
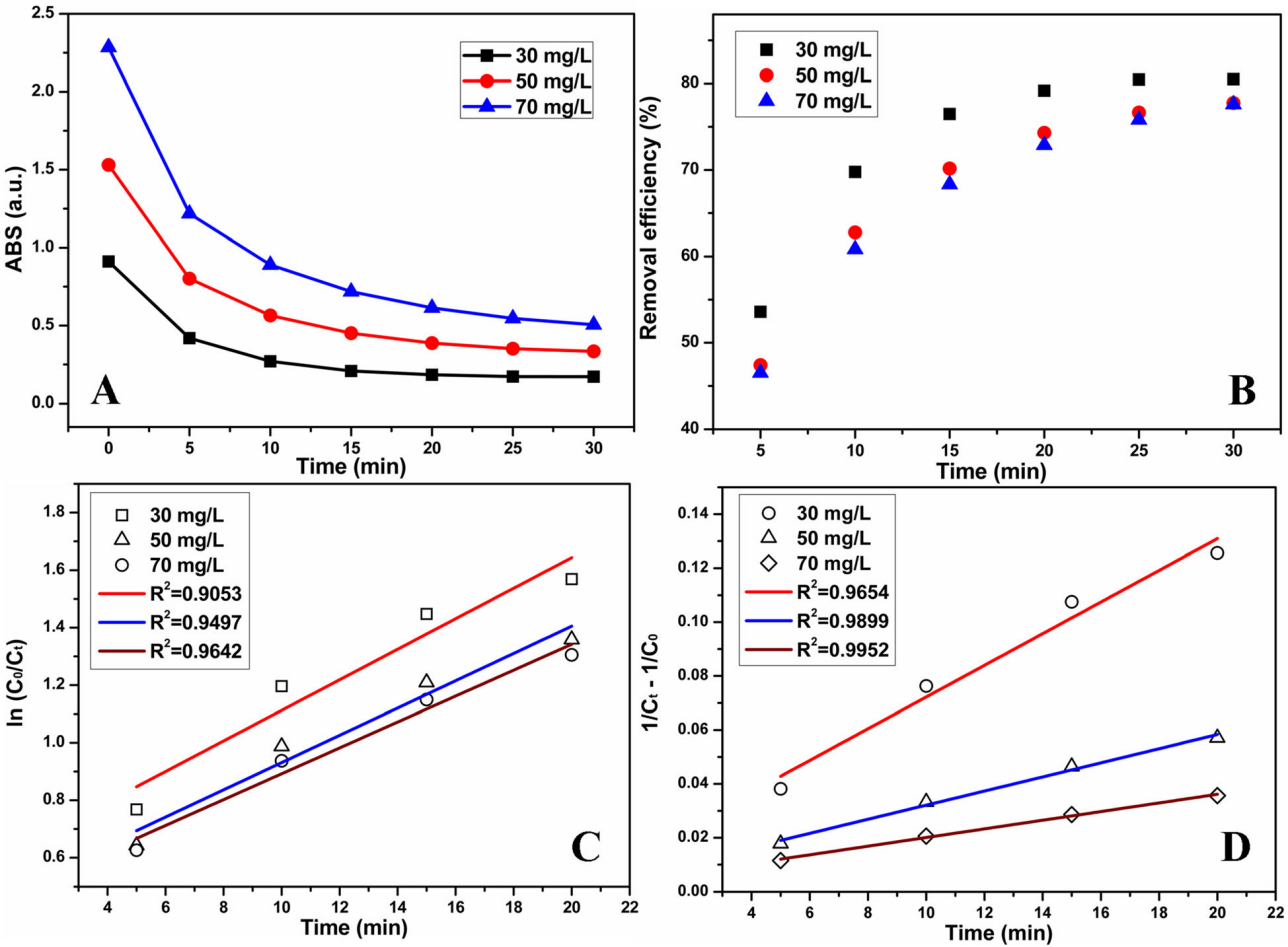
The time-dependent **a** absorption intensity of TCH and **b** removal efficiency for the degradation of different initial concentrations of TCH. **c** Linear first-order kinetic plots and **d** linear second-order kinetic plots for degradation of different initial concentrations of TCH. (Conditions: a dose of MnO_2_ NPs/ESM was 0.1740 g/L, controlled pH was 3.0)

To further understand the TCH degradation by MnO_2_ NPs/ESM, the kinetic study was carried out by changing the TCH initial concentration or dose of MnO_2_. We investigated the kinetics of degradation by different amounts of MnO_2_ under buffered conditions. Figure 6a displayed the time-dependent absorption intensity of TCH degraded by different doses of MnO_2_ (0.0960, 0.1440, and 0.1920 g/L), and the corresponding removal efficiencies were calculated in Fig. 6b. And linear kinetic plots by pseudo-first-order and pseudo-second-order were fitted in Fig. 6c, d, respectively. Moreover, degradation at different initial concentrations of TCH (30, 50, and 70 mg/L) with buffer was studied through monitoring the absorption intensity (Fig. 7a) and removal efficiency (Fig. 7b) at different times. Figure 7c, d fitted the linear first/second-order kinetic plots to investigate the kinetics. Similarly, degradation kinetics at different amounts of MnO_2_ NPs and different initial TCH concentrations under unbuffered conditions were studied in Additional file 8: Figure S8 and Additional file 9: Figure S9, respectively. Table 1 exhibited the kinetic data obtained from different conditions. The correlation coefficients were linear-fitted and calculated to demonstrate the kinetic of TCH degradation by MnO_2_ NPs/ESM. Generally, the correlation coefficients calculated through the pseudo-second-order model were higher than that through pseudo-first-order, indicating this process was more consistent with the pseudo-second-order model. In detail, this pseudo-second-order model had higher correlation coefficients at small doses of MnO_2_ or high initial concentrations of TCH. And either way, correlation coefficients were closer to 1 in the buffered conditions compared with the degradation without a buffer.

**Table 1:** The kinetic data under different conditions

Initial conc.^a^	Dose^b^	Buffer^c^	*R*^2^ (first-order)	*R*^2^ (second-order)
50	0.0960	Yes	0.9773	0.9968
50	0.1440	Yes	0.9762	0.9982
50	0.1920	Yes	0.9623	0.9944
30	0.1920	Yes	0.9053	0.9654
50	0.1920	Yes	0.9497	0.9899
70	0.1920	Yes	0.9642	0.9952
50	0.0960	No	0.9637	0.9841
50	0.1440	No	0.8719	0.9126
50	0.1920	No	0.8558	0.9053
30	0.1920	No	0.8775	0.9330
50	0.1920	No	0.8558	0.9053
70	0.1920	No	0.8896	0.9249

^a^Initial concentration of TCH (mg/L)

^b^Dose of MnO_2_ (g/L)

^c^PBS buffer (pH = 3.0)

### Comparison of Commercial MnO_2_ Powder and Other Reported Materials

**Fig. 8:**
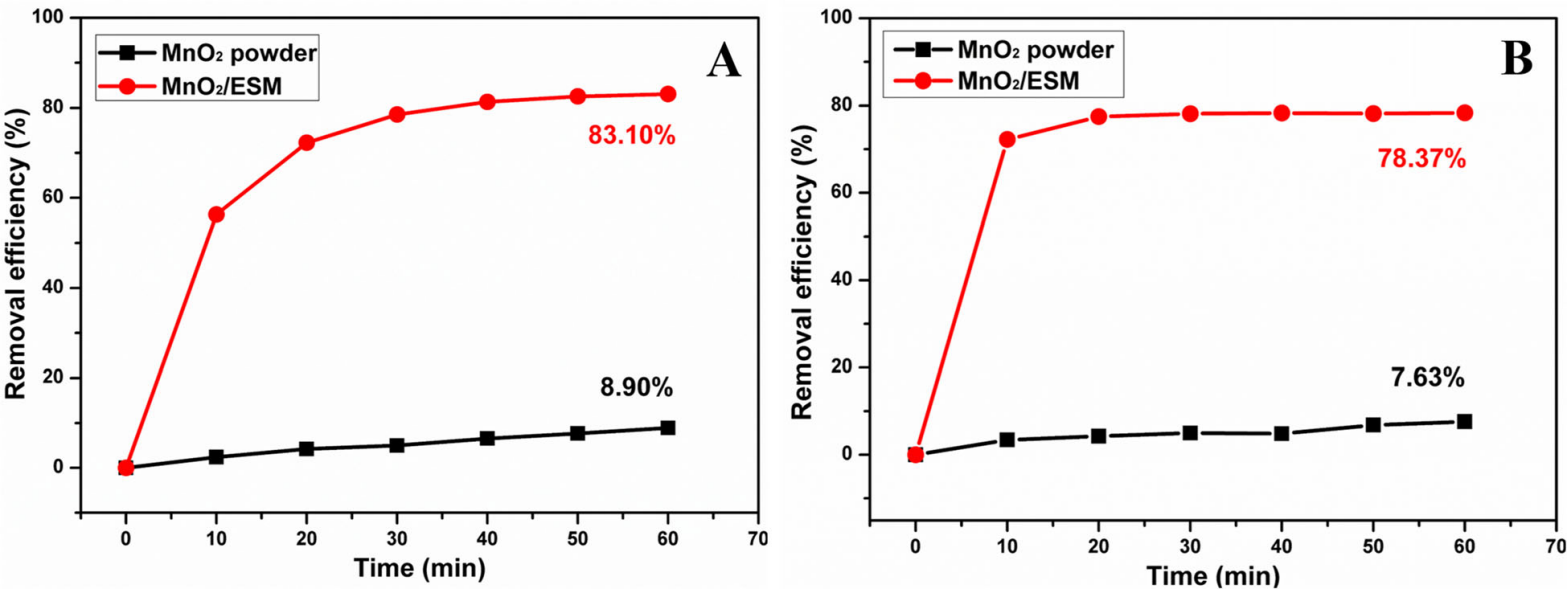
Comparison of the removal efficiency of TCH by equal commercial MnO_2_ powder and MnO_2_ NPs/ESM under **a** buffered and **b** unbuffered conditions

To illustrate the advanced property of as-prepared MnO_2_ NPs/ESM, the equal amount of commercial MnO_2_ powder was contrastively tested for TCH decontamination under the same conditions. Figure 8 showed the removal efficiency from as-prepared MnO_2_ NPs/ESM and commercial MnO_2_ powder with or without a buffer. It was indicated that MnO_2_ NPs/ESM showed a prominent advantage over commercial MnO_2_ powder under both conditions. Though removal efficiency of around 80% through MnO_2_ decontamination was obtained in previous work [38, 39], it could reach up to 98% under pH = 6.5 through a MnO_2_ and zero-valent iron (ZVI)-based permeable reactive barrier (PRB) system [56], which was attributed to the multiple effects from ZVI coupling with MnO_2_. Besides, other materials were also applied to TC decontamination. Immobilized TiO_2_ nanobelts modified by Au and CuS nanoparticles (Au–CuS–TiO_2_ NBs) displayed a removal efficiency of 96% towards oxytetracycline (OTC) owing to their superior photocatalytic activity [57]. Graphene oxide (GO) as an efficient adsorbent showed a good removal for TC after 24 h (*R* = 96%) [58]. Powder activated carbon/Fe_3_O_4_ magnetic nanoparticles (PAC/Fe_3_O_4_ MNPs) were applied as a catalyst to H_2_O_2_-assisted TC degradation, and removal efficiency of 94.5% was obtained [59]. It was noticed that the removal efficiency could be enhanced by prolonging the treatment time or increasing the material doses [39]. Nevertheless, all the work needs complicated degradation measurement and subsequent processing which increase the labor and test time. The handy operation of our method such as neither centrifugation nor filtration would facilitate the decontamination procedure.

## Conclusions

MnO_2_ nanoparticles were synthesized in this work through a super easy procedure by mixing the eggshell membrane and potassium permanganate solutions. This harsh reaction conditions or complicated aftertreatment needless method made synthesis and purification process quickly and handy. The obtained MnO_2_ nanoparticles dispersed uniformly onto the surface of fibrous proteins to form a microcosmic/macroscopic combination mode. Further, the eggshell membrane-templated MnO_2_ nanoparticles were applied to tetracycline hydrochloride decontamination. A removal efficiency of 83.10% after 60 min under the buffered condition and pseudo-second-order model kinetics were obtained. Most notably, MnO_2_ NPs/ESM can be separated easily by taking it out of the solutions, which avoided complex operation like centrifugation or filtration, making it an advantage in nanomaterial-based wastewater decontamination.

## Additional files

Additional file 1: **Figure S1.** Size distribution of as-prepared MnO_2_ NPs. (TIF 168 kb)

Additional file 2: **Figure S2.** (A) The photos of MnO_2_ NPs/ESM before and after NaOH treatment. (B) The photos of filtrated solutions after NaOH treatment from blank ESM and MnO_2_ NPs/ESM, respectively. (C) Size distribution of ESM after NaOH treatment. (TIF 1576 kb)

Additional file 3: **Figure S3.** Contrast pictures of MnO_2_ NPs/ESM (A) before reaction, (B) right after immersed into GSH aqueous solution (1 mM) and (C) after 1 min soaking. (TIF 1310 kb)

Additional file 4: **Figure S4.** The TG curves of ESM (black) and MnO_2_ NPs/ESM (red). (TIF 501 kb)

Additional file 5: **Figure S5.** FTIR spectra of ESM and MnO_2_ NPs/ESM with deconvolution. (TIF 1223 kb)

Additional file 6: **Figure S6.** Linear calibration plot for TCH ranging from 0.1 to 75 mg/L without a buffer. (TIF 165 kb)

Additional file 7: **Figure S7.** Linear calibration plot for TCH ranging from 0.1 to 75 mg/L with buffer (pH = 3). (TIF 1673 kb)

Additional file 8: **Figure S8.** Degradation kinetics of TCH at different amounts of MnO_2_ NPs/ESM under unbuffered conditions. The time-dependent of absorption intensity of TCH (A), removal efficiency by different amounts of MnO_2_ NPs/ESM treatment (B), linear first order kinetic plots (C) and linear second order kinetic plots (D) with different amounts of MnO_2_ NPs/ESM treatment. (conditions: initial concentration of TCH was 50 mg/L, without PBS buffer.). (TIF 4052 kb)

Additional file 9: **Figure S9.** Degradation kinetics of TCH at different initial TCH concentration under unbuffered conditions. The time-dependent of absorption intensity of TCH (A) and (B) removal efficiency for degradation of different initial concentrations of TCH. (C) Linear first order kinetic plots and (D) linear second order kinetic plots for degradation of different initial concentrations of TCH. (conditions: dose of MnO_2_ NPs/ESM was 0.1740 g/L, without PBS buffer.). (TIF 860 kb)

## Abbreviations

ESM: Eggshell membrane
GSH: Glutathione
HRTEM: High-resolution transmission electron microscopy
NPs: Nanoparticles
OTC: Oxytetracycline
PBS: Phosphate buffer saline
PPCPs: Pharmaceuticals and personal care products
SEM: Scanning electron microscopy
TCH: Tetracycline hydrochloride
TCs: Tetracyclines
TEM: Transmission electron microscopy
TG: Thermogravimetry
UV-vis: Ultraviolet-visible
XPS: X-ray photoelectron spectroscopy
