# Supplementary figures and images for: Eggshell Membrane-Templated MnO2 Nanoparticles: Facile Synthesis and Tetracycline Hydrochloride Decontamination
Source: Nanoscale Res Lett. 2018 Aug 28;13:255. doi: 10.1186/s11671-018-2679-y (PMC6113195; doi:10.1186/s11671-018-2679-y)

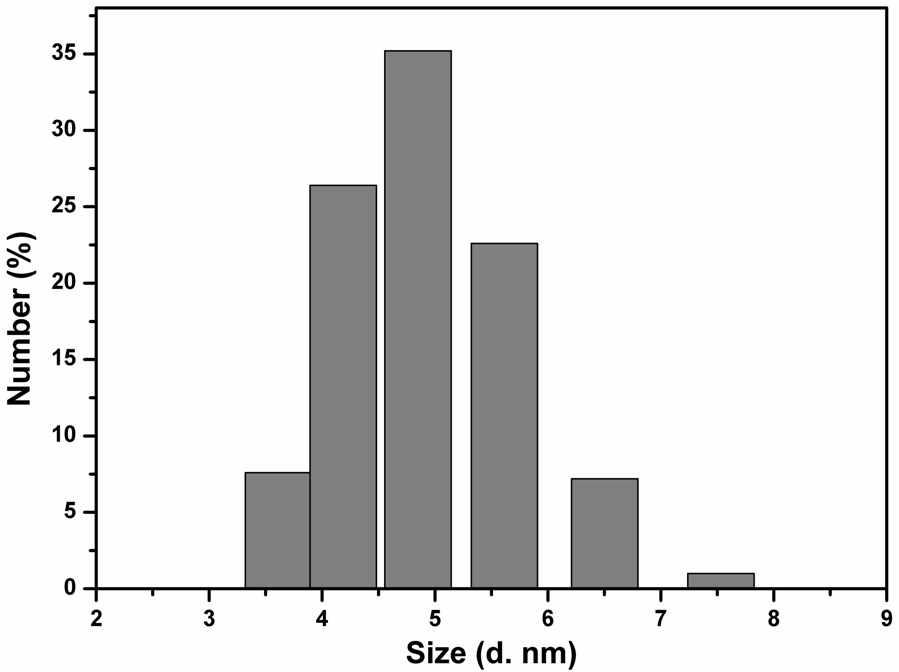

Supplement: Supplementary file 1 — Figure S1. Size distribution of as-prepared MnO2 NPs. (TIF 168 kb) [file 11671_2018_2679_MOESM1_ESM.tif]

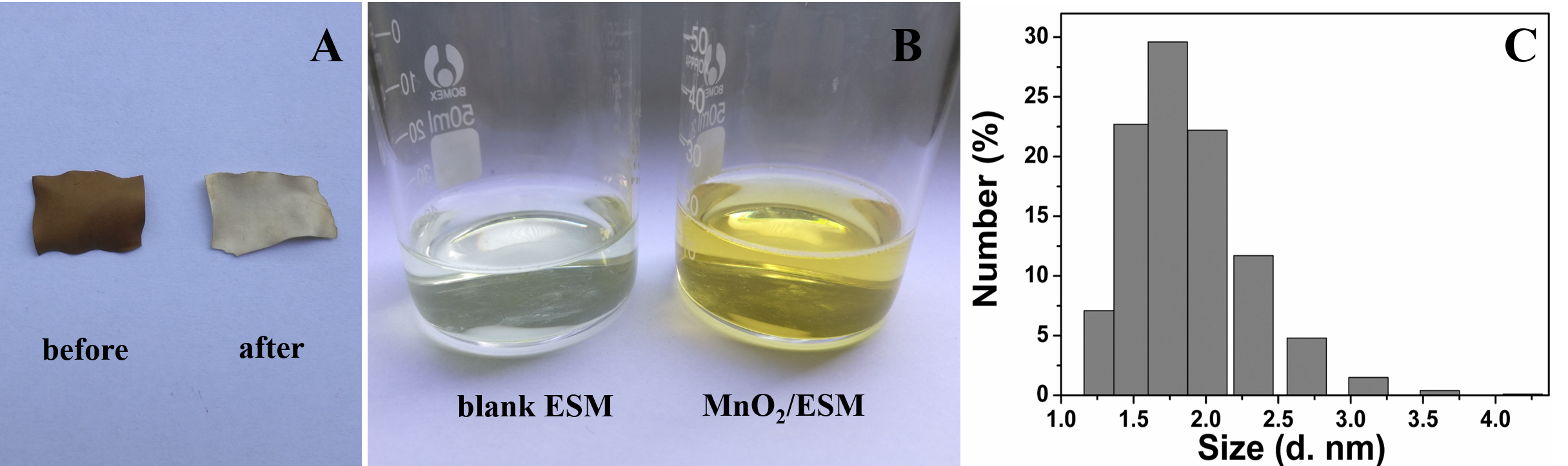

Supplement: Supplementary file 2 — Figure S2. (A) The photos of MnO2 NPs/ESM before and after NaOH treatment. (B) The photos of filtrated solutions after NaOH treatment from blank ESM and MnO2 NPs/ESM, respectively. (C) Size distribution of ESM after NaOH treatment. (TIF 1576 kb) [file 11671_2018_2679_MOESM2_ESM.tif]

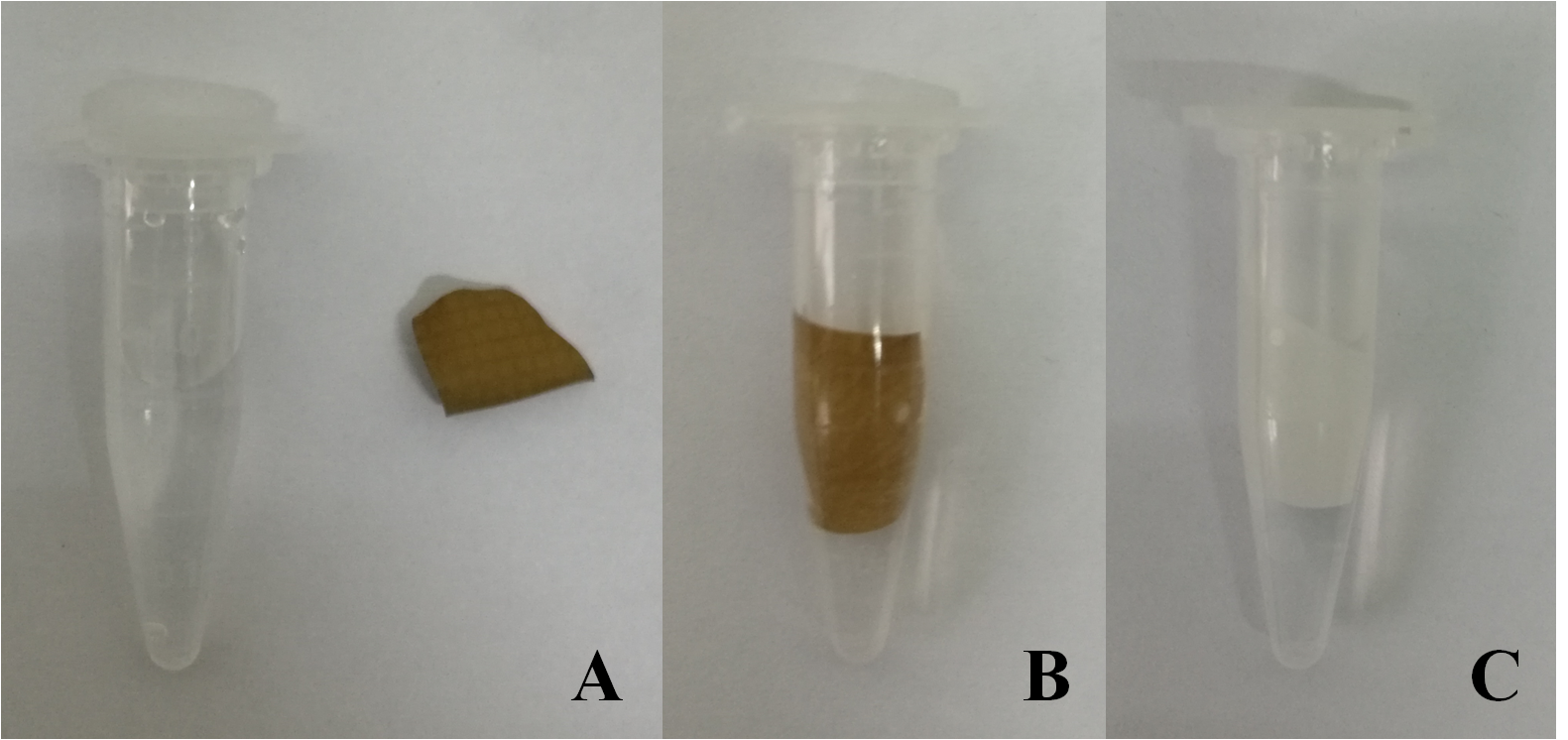

Supplement: Supplementary file 3 — Figure S3. Contrast pictures of MnO2 NPs/ESM (A) before reaction, (B) right after immersed into GSH aqueous solution (1 mM) and (C) after 1 min soaking. (TIF 1310 kb) [file 11671_2018_2679_MOESM3_ESM.tif]

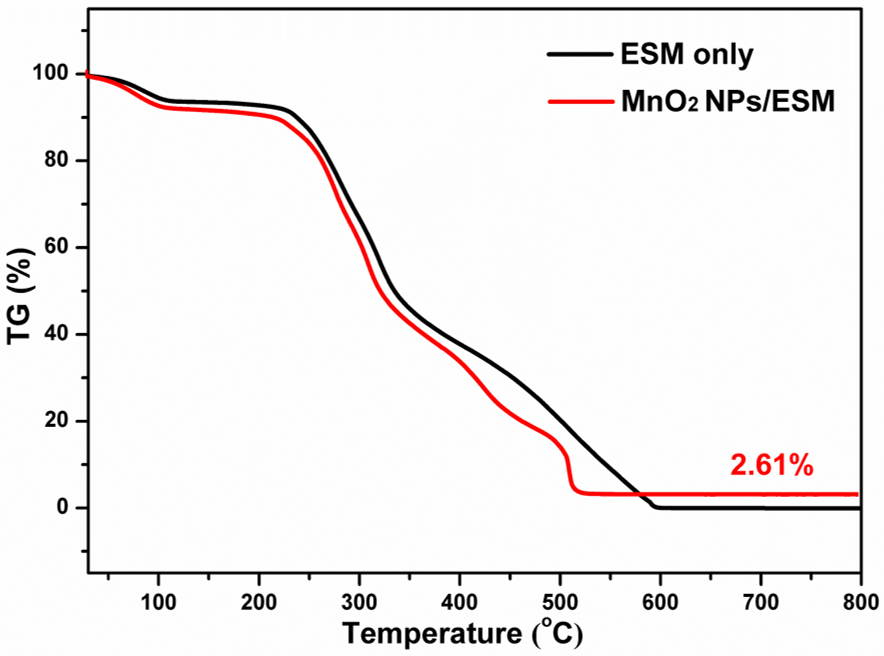

Supplement: Supplementary file 4 — Figure S4. The TG curves of ESM (black) and MnO2 NPs/ESM (red). (TIF 501 kb) [file 11671_2018_2679_MOESM4_ESM.tif]

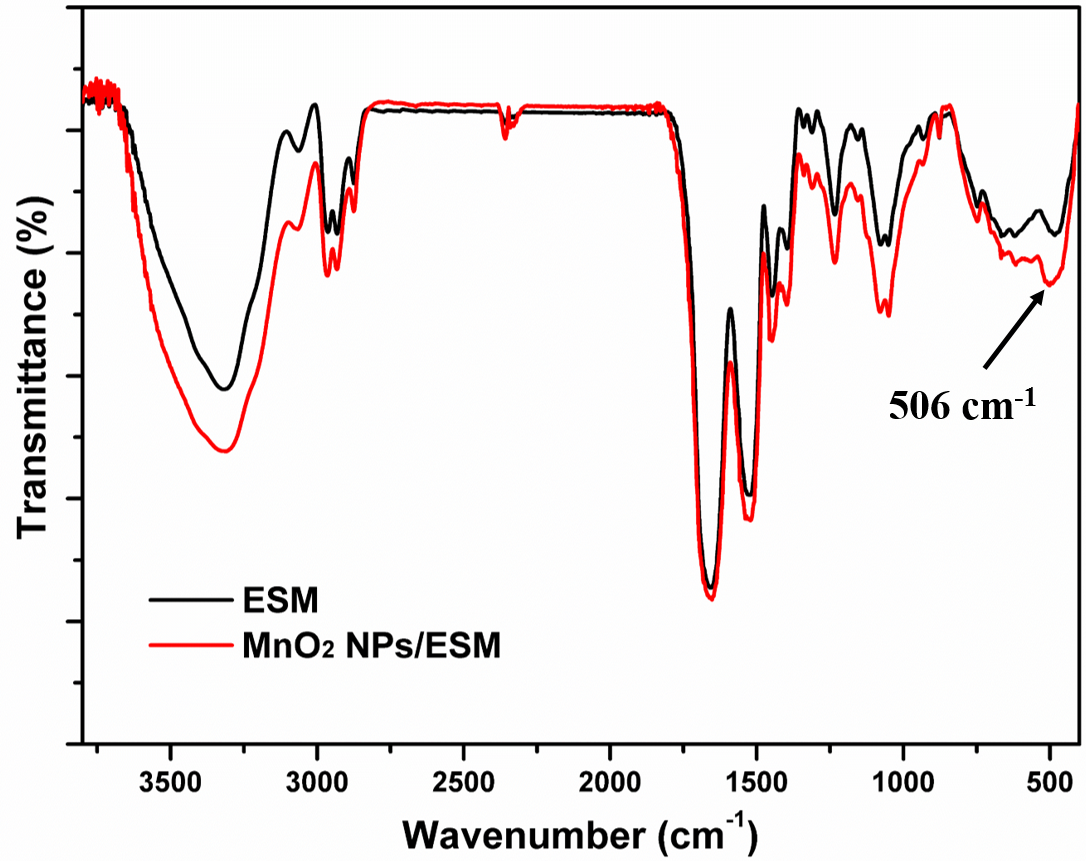

Supplement: Supplementary file 5 — Figure S5. FTIR spectra of ESM and MnO2 NPs/ESM with deconvolution. (TIF 1223 kb) [file 11671_2018_2679_MOESM5_ESM.tif]

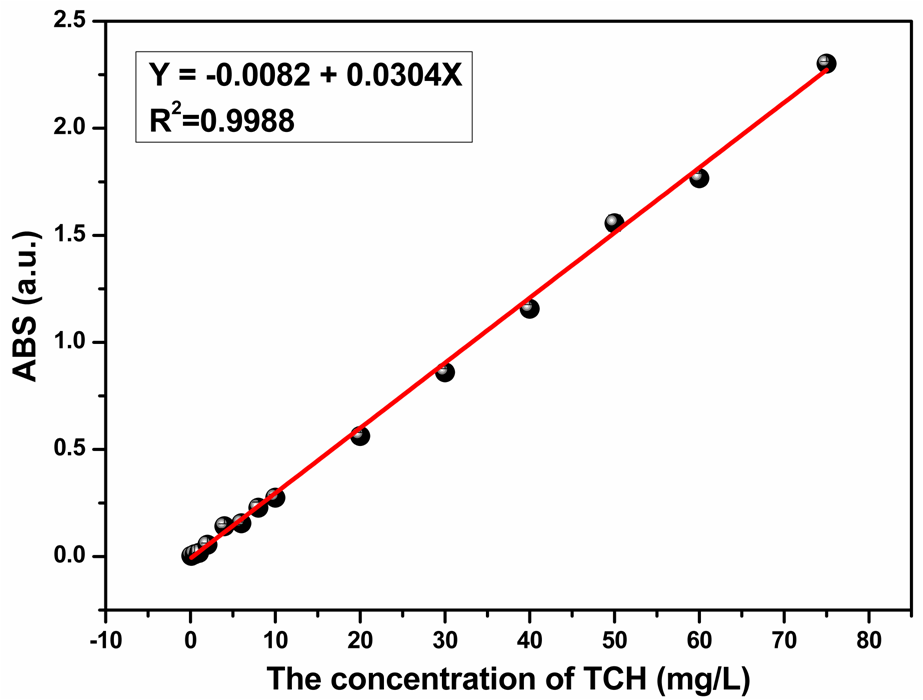

Supplement: Supplementary file 6 — Figure S6. Linear calibration plot for TCH ranging from 0.1 to 75 mg/L without a buffer. (TIF 165 kb) [file 11671_2018_2679_MOESM6_ESM.tif]

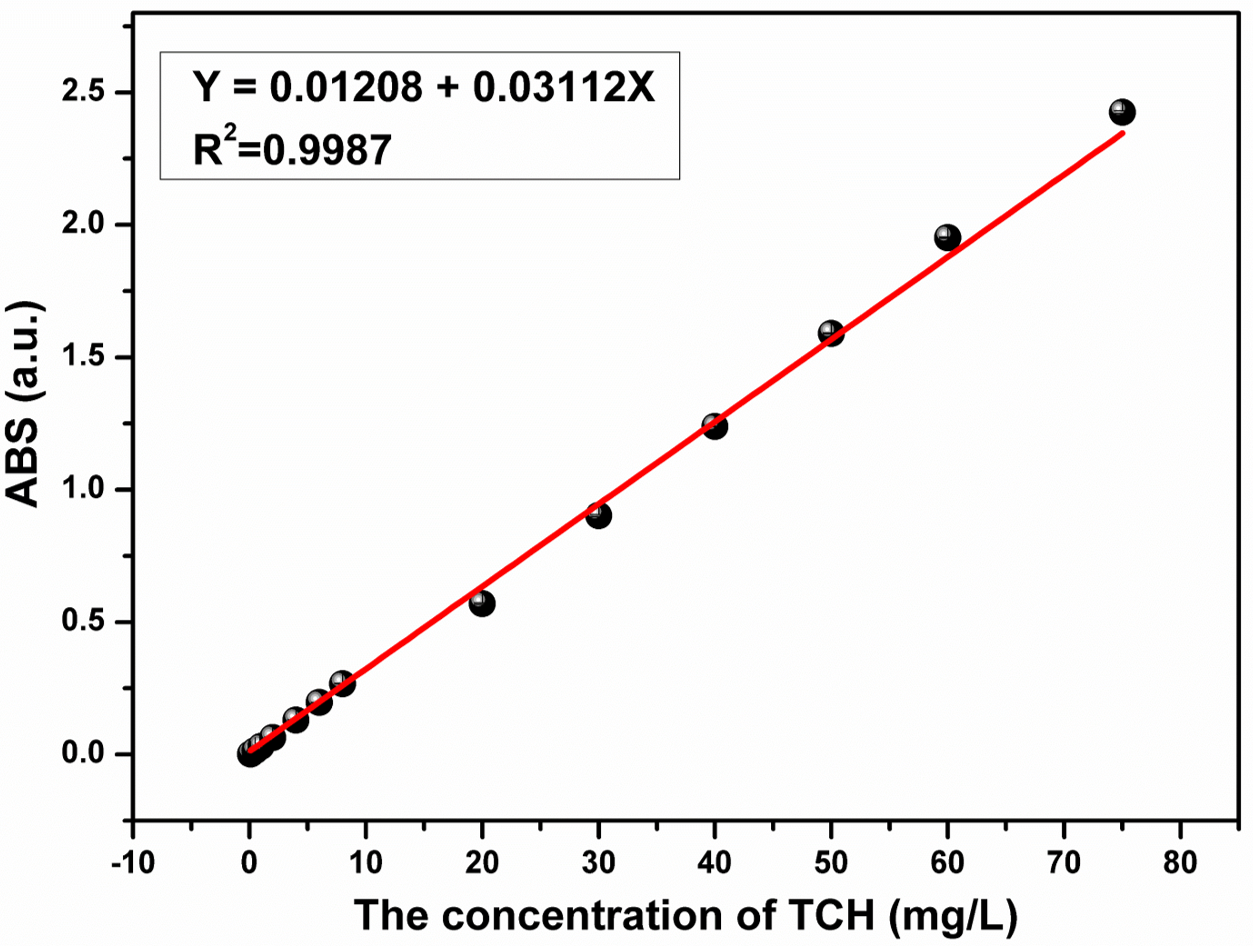

Supplement: Supplementary file 7 — Figure S7. Linear calibration plot for TCH ranging from 0.1 to 75 mg/L with buffer (pH = 3). (TIF 1673 kb) [file 11671_2018_2679_MOESM7_ESM.tif]

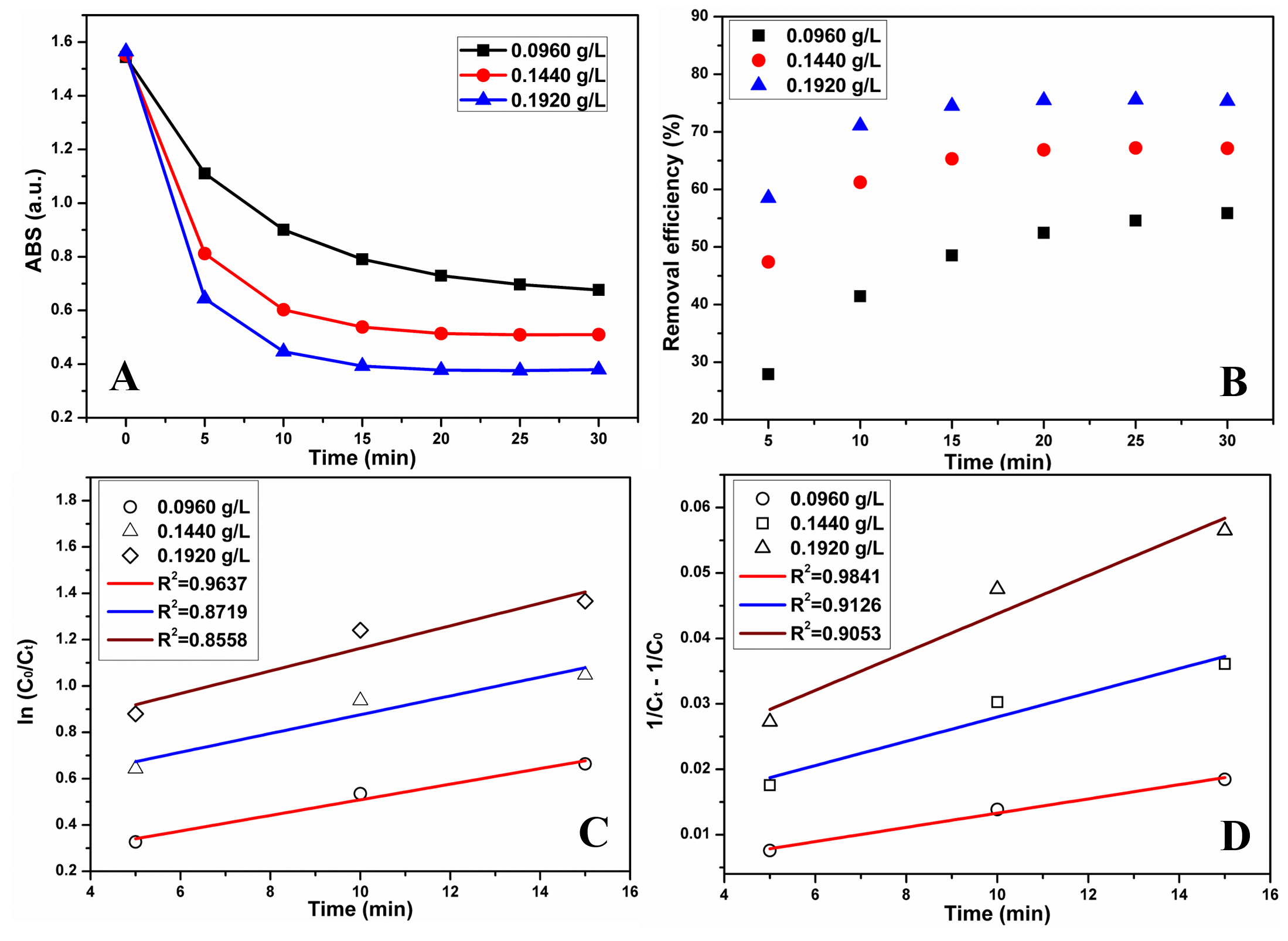

Supplement: Supplementary file 8 — Figure S8. Degradation kinetics of TCH at different amounts of MnO2 NPs/ESM under unbuffered conditions. The time-dependent of absorption intensity of TCH (A), removal efficiency by different amounts of MnO2 NPs/ESM treatment (B), linear first order kinetic plots (C) and linear second order kinetic plots (D) with different amounts of MnO2 NPs/ESM treatment. (conditions: initial concentration of TCH was 50 mg/L, without PBS buffer.). (TIF 4052 kb) [file 11671_2018_2679_MOESM8_ESM.tif]

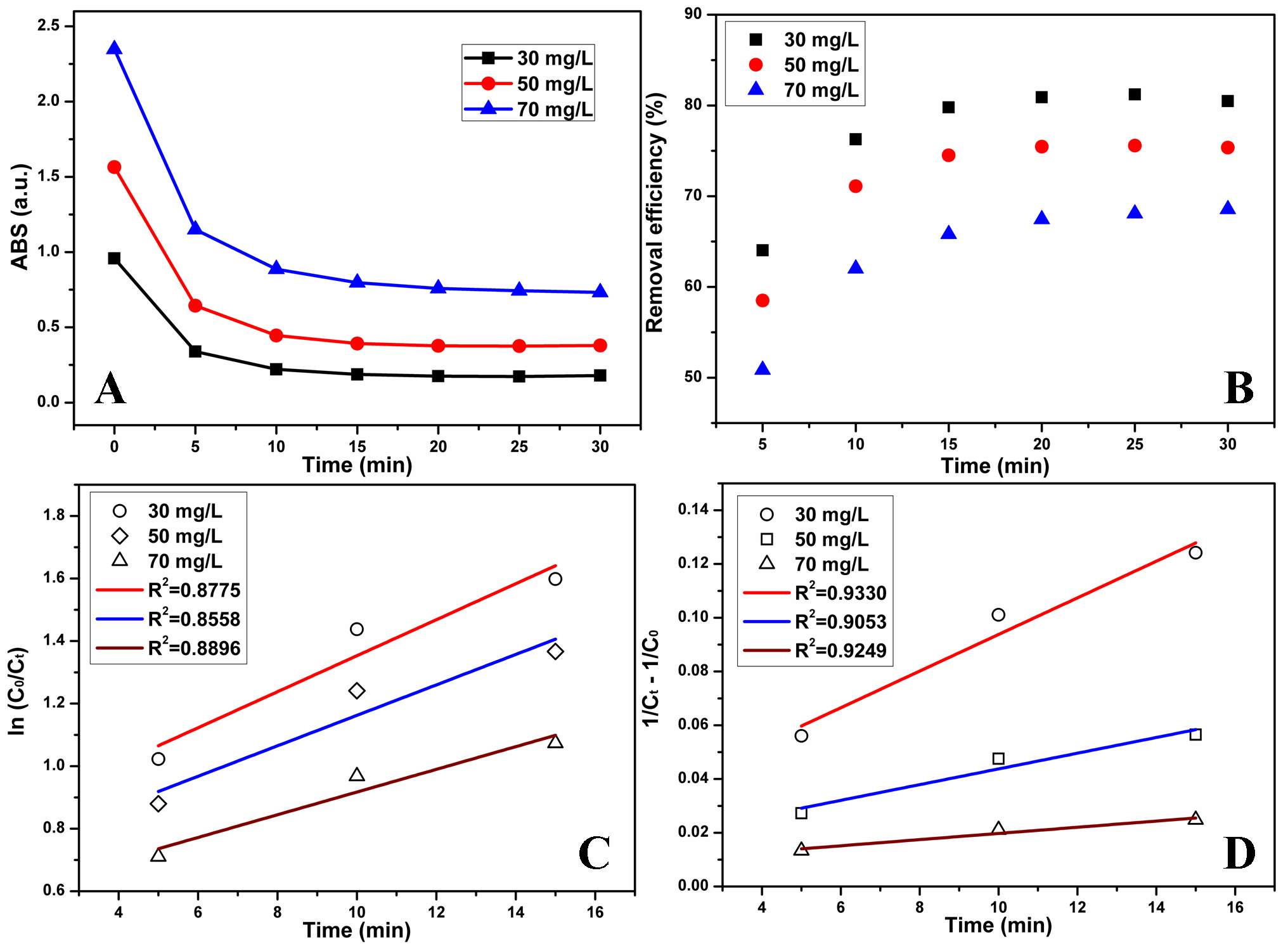

Supplement: Supplementary file 9 — Figure S9. Degradation kinetics of TCH at different initial TCH concentration under unbuffered conditions. The time-dependent of absorption intensity of TCH (A) and (B) removal efficiency for degradation of different initial concentrations of TCH. (C) Linear first order kinetic plots and (D) linear second order kinetic plots for degradation of different initial concentrations of TCH. (conditions: dose of MnO2 NPs/ESM was 0.1740 g/L, without PBS buffer.). (TIF 860 kb) [file 11671_2018_2679_MOESM9_ESM.tif]
